# Early Closure of Gastroschisis After Silo Placement Correlates with Earlier Enteral Feeding

**Published:** 2015-07-01

**Authors:** Jamie Harris, Jennifer Poirier, Debra Selip, Srikumar Pillai, Ami N. Shah, Carl-Christian Jackson, Bill Chiu

**Affiliations:** 1 Division of General Surgery, Rush University Medical Center. 1653 W. Congress Parkway Jelke Suite 792, Chicago; 2 Rush Fetal and Neonatal Medicine Center, Rush Children's Hospital. 1653 W Congress Pkwy 622 Murdock, Chicago; 3 Division of Pediatric Surgery, Rush University Medical Center. 1653 W. Congress Parkway Jelke Suite 792, Chicago; 4 Division of Pediatric Surgery, Floating Hospital for Children - Tufts Medical Center, 800 Washington Street, Boston

**Keywords:** Closure timing, Gastroschisis, Enteral feeding, Silo

## Abstract

Objectives: Gastroschisis is a congenital anomaly affecting 2.3-4.4/10,000 births. Previous studies show initiation of early enteral feeds predicts improved outcomes. We hypothesize that earlier definitive closure after silo placement; can lead to earlier enteral feed initiation.

Design/ Setting/ Duration: Retrospective review of patients with gastroschisis from 2005 and 2014 at a single institution.

Material and Methods: The data, including ethnicity, gestational age, birth weight, time to definitive closure, and time of first and full feeds, were analyzed using both Spearman’s rho and the Kruskal-Wallis rank sum test where appropriate; a p value less than 0.05 was considered significant.

Results: Forty-three patients (24 males, 19 females) born with gastroschisis were identified. Overall survival rate was 88% (38/43). Forty of the 43 patients had a silo placed prior to definitive closure. Median days to closure were 6 (0 to 85) days. First feeds on average began on day of life (DOL) 17, and full feeds on DOL 25. Earlier closure of gastroschisis correlated with early initiation of feeds (p=0.0001) and shorter time to full feeds (p=0.018), closure by DOL4 showed a trend toward earlier feeding (p=0.13).

Conclusion: Earlier closure of gastroschisis after silo placement was associated with earlier feed initiation and shorter time to full feeds.

## INTRODUCTION

Gastroschisis is a defect in the abdominal wall that results in abdominal contents, most commonly intestines, herniating through the defect. The incidence of gastroschisis has increased from 2.3/10,000 live births in 1995 to 4.4/10,000 in 2005 [1]. Gastroschisis occurs with a higher frequency in younger mothers, and among women with lower socioeconomic status [2]. When compared with other abdominal wall defects, namely omphalocele, gastroschisis has an improved overall survival, around 90%. However, there is morbidity associated with gastroschisis including prolonged neonatal intensive care stay, use of total parental nutrition (TPN), and infections due to prolong intravenous access [3].

Previous studies have examined predictors of outcomes in management with patients with gastroschisis. Factors that predict favorable outcomes include increased birth weight, simple gastroschisis, defined as those with intact bowel, without evidence of perforation, necrosis, or atresia, and the time to initiation of oral feeds [3-6]. The patients who began feeding earlier were shown to have shorter duration of TPN, length of hospital stay, and infectious complication [6]. Initiation of feeds in gastroschisis is based on clinical markers including: definitive closure, resolution of abdominal distention, nasogastric output becoming nonbilious, and stooling [6].

Current strategies for gastroschisis closure include early repair of fascial defect on day of life zero, or delayed fascial closure after placement of abdominal content within a preformed silo followed by serial reduction. It has been shown that outcomes are improved with silo placement and serial reductions, including decreased incidence of necrotizing enterocolitis as well as closure related complications, including ability to close primarily and abdominal compartment syndrome [7, 8]. While this method of closure has become more popular, ideal timing of definitive closure after silo placement is still being investigated. We hypothesize that earlier definitive closure of the gastroschisis defect after silo placement, thereby minimizing intestinal irritation, can lead to earlier progression to enteral feeding.

## MATERIALS AND METHODS

After receiving Internal Review Board approval (#14072108-IRB01), a retrospective chart review of all infants born with gastroschisis, as well as their mothers, treated at a single institution from the years 2005-2014 was done. Obstetric notes, prenatal ultrasounds and hospital charts were reviewed.

Maternal data examined included maternal age and race. Infant data collected included whether bowel dilation was present on prenatal ultrasound, route of delivery and reasoning if via cesarean section, APGAR scores, sex, gestational age at birth, length of hospital stay, and mortality. Simple versus complex gastroschisis, defined as the presence of intestinal atresia, ischemia, or perforation was noted. Hospital course data including ventilation strategies, days on the ventilator, days from birth at which first feedings were initiated, and when the infant reached full feeds were also obtained. Enteral feeds at our institution are started based on signs of return of bowel function, including stooling, decreased orogastric tube output, and decreased abdominal distention. Feeds are slowly advanced based on tolerance, lack of emesis or abdominal distention, and based on clinical judgment by surgeon and neonatologist. Full enteral feeds are defined as receiving the total caloric need of the infant enterally. Operative data, including date of initial closure or preformed silo placement, and subsequent operations were noted. Silo placement was based on surgeon preference, amount of intestines involved, and degree of inflammation and irritation noted around the bowel, all silos placed were preformed silos. Definitive closure was done using suture to close the defect.

The data were analyzed using t-tests, Spearman’s rho, Fisher’s exact tests, Wilcox Mann Whitney test, and the Kruskal-Wallis rank sum test where appropriate. A p value of less than 0.05 was considered significant.

## RESULTS

Forty-three infants with the diagnosis of gastroschisis were identified. Of those, 24 were male, and 19 were female. The diagnosis of gastroschisis was made on prenatal ultrasound for 41 out of 43 patients, and there were seven cases of complex gastroschisis. Overall survival rate was 88% (38/43), and two of the mortalities involved complex gastroschisis, one had midgut volvulus with subsequent necrosis, one with necrotizing enterocolitis, and one born with pulmonary hypoplasia and severe pulmonary hypertension who expired on day of life 0. The median hospitalization was 41 days (0-498 days). Median maternal age was 20 years (15 - 33 years), with a distribution of maternal race as 35% Hispanic, 33% Caucasian, 30% African American, and 2% American Indian. There were 25 cesarean sections in the group. Reasons for C-section varied, but include increased fetal bowel dilatation, variable decelerations, failure to progress, or multiple gestations. On prenatal ultrasound, bowel dilatation was appreciated in 22 fetuses. APGAR scores on average at 1 minute and 5 minutes were 6.93 and 8.31, respectively. The median number of ventilator days was 3 days, 0-85 days. Oxygenation strategies included nasal cannula, high flow nasal cannula, conventional ventilator, and oscillator.

Median gestation age at delivery was 35 1/3 weeks (28-39 weeks). When patients were separated into those born before 37 weeks and those after, the two groups did not significantly differ on time to first feeds (Wilcox Mann Whitney test, p=1). The median time to first feed for the 37weeks or greater gestational age was 18 days, and the median time to first feed for the less than 37 weeks gestation was 16 days. The two groups also did not significantly differ on mortality (Fisher’s exact test, p=0.1957). (Figure 1)

**Figure:**
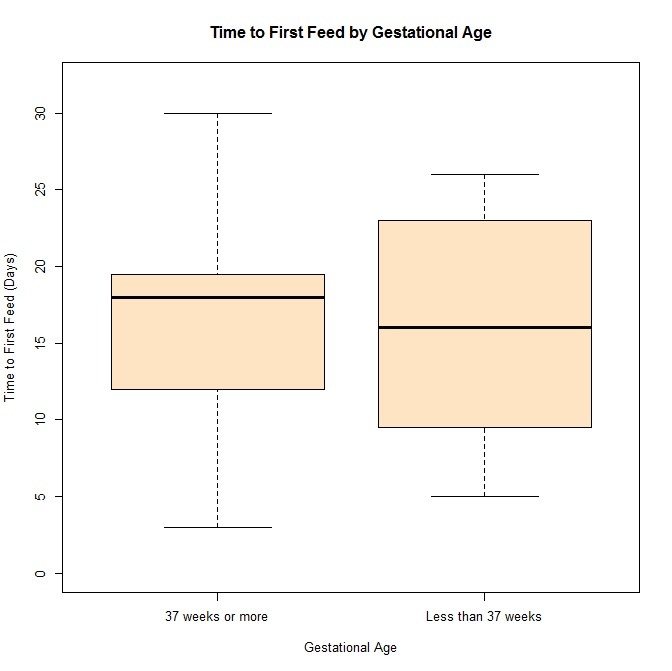
Figure 1: Relationship between time to enteral feeds and gestational age less than 37 weeks or greater than 37 weeks at delivery. No difference in time to feed was found between the two groups.

Median birth weight was 2050 grams (740 - 3482 grams). This did not correlate with the time to definitive closure of the gastroschisis (Spearman’s rho, p=0.6). When analyzing the simple group alone to ensure there was not bias from inclusion of complex cases, it again did not correlate with the simple gastroschisis group alone (Spearman’s rho, p=0.39). There was no association between time to first feeding and birth weight in the total cohort (Spearman’s rho, p=0.52) or in the simple gastroschisis group (Spearman’s rho, p=0.46). Additionally, time to first feeds was not significantly different among different ethnic groups (Kruskal Wallis test, p=0.42-0.68). 

Five patients were closed on day of life (DOL) 0. Of those closed on DOL 0, two underwent silo placement with definitive closure later the same day, one with immediate closure, one with resection for necrosis from midgut volvulus that appeared chronic at the time of delivery, and one with colonic atresia, colostomy and closure. Median days to closure were 6 (0-85). First feeds, on average, began on DOL 17. Earlier definitive closure of all patients with gastroschisis was correlated with early initiation of feeds (Spearman’s rho=0.65, p=0.0001). Based on previous studies showing worse outcomes in patients with complex gastroschisis, those with simple gastroschisis alone were analyzed separately to eliminate any potential confounding factors from complex gastroschisis [4,5]. Additionally, all patients in the simple gastroschisis group had silos placed. Again, a correlation was demonstrated between earlier closure after silo placement and earlier feeds in the simple alone gastroschisis group (Spearman’s rho=0.69, p less than 0.0001). Full enteric feeds were achieved on average by DOL 25. Shorter time to full feeds as related to timing of definitive closure for the entire cohort (Spearman’s rho=0.46, p=0.018) as well as for the simple group alone (Spearman’s rho= 0.42, P=0.04) was also demonstrated (Figure 2, Table 1).

**Figure:**
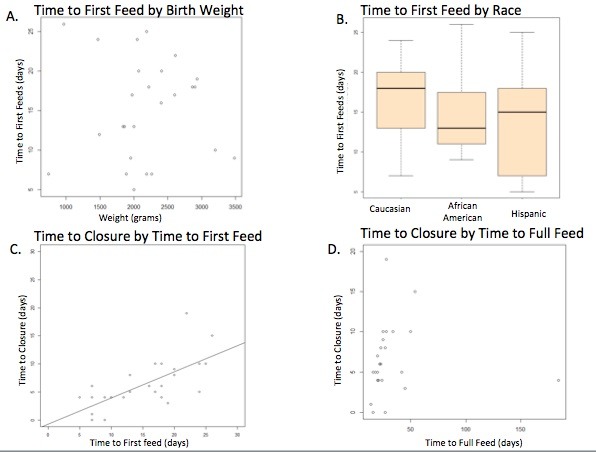
Figure 2: Relationship between birth weight, ethnicity and closure with initiation of enteral feeds. No correlation was seen (A) between birth weight and feed initiation or (B) between ethnicity and feed initiation. Earlier definitive closure of gastroschisis defect correlates with (C) earlier initiation of enteral feeds and (D) attainment of full enteral feeds.

**Figure:**
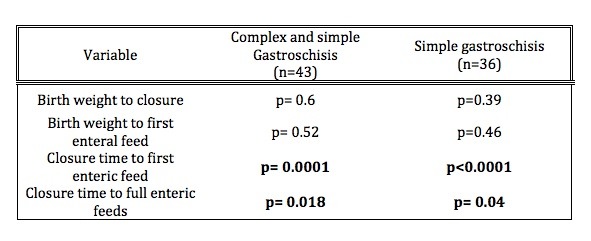
Table 1: Correlation of variables between entire cohort of gastroschisis, as well as simple gastroschisis only with time to closure as well as time to enteral feeding.

Finally, within the simple gastroschisis group alone, cohorts were compared to determine when ideally the defect should be definitively closed. To prevent the confounding effect of time to closure on time to feeds, the time of first feeds from the day of closure was used as the outcome measure in this analysis. There was no difference between the two groups (t-test, t=-0.63, p=0.53) when closed at or before DOL5 when compared with those closed after. Mean days until first feed from closure were 8.7 at or before 5 days, and 9.8 after 5 days. However, there did appear to be a trend toward earlier feeds when closed on day of life 4. For those closed by 4 days feeds were initiated 7.5 days after closure, and for those after 10.3 days (t-test= -1.58, p=0.13).

## DISCUSSION

Gastroschisis is a neonatal disease that can be associated with prolonged hospitalizations, TPN, prolonged ventilator time, infection, and sepsis related to prolonged TPN; moreover, the incidence of gastroschisis is increasing [9,10]. Many different aspects of prenatal and perinatal care have been assessed to determine their influence on outcome. We found that birth weight, ethnicity and gestational age did not correlate with initiation of feeding. However, we did find that earlier definitive closure after silo placement was related to earlier initiation of enteral feeding as well as full enteral feeds. 

When comparing our cohort of patients to those from previous studies, one major difference was in the ethnicity of patients. Previous studies reported Caucasian and Hispanic women as being the most common ethnicities with gastroschisis, with lowest rates in African Americans [2]. We found similar numbers of Caucasian, Hispanic, and African American patients in our cohort, a reflection of the inner city population served by our hospital. However, even with this ethnicity distribution, we found that we had other similar patient characteristics and outcomes compared to those reported in the literature, such as a young maternal age on average 19.5 years, 20-24 years, Rasmussen et al., and a complex gastroschisis rate of 16%, 11% in Baerg et al. [2, 11]. It is possible that due to the smaller sample size of the group our mortality rate is higher than other published outcomes, ranging from 90-95%, Overcash et al. [10].

Aljahdali et al. found that earlier enteral feeding after closure was associated with better overall outcome [6]. These authors found that patients who started enteral feeding within 0-7 days after closure had significantly shorter length of hospital stay, fewer number of days on TPN, and fewer infectious complications when compared with those that started 21 days after closure. Bucher et. al. found that time to closure was associated with earlier age of full enteric feeding and less TPN duration [12]. Banyard also identified decreased TPN and earlier enteral feeds with those closed primarily compared with silo closure [13]. Our data showed that earlier closure of the gastroschisis defect after silo placement correlated with earlier initiation of and progression to full enteric feeds, suggesting that earlier closure could lead to improved outcome. 

One possible mechanism for the better outcome with early closure after preformed silo placement is the decreased irritation and inflammation of the bowel when less time is spent outside of the abdomen. It has been well described in literature that the in utero exposure to amniotic fluid leads to bowel inflammation and a fibrous coating [14,15]. Burc et al. found elevated ferritin and proteins in fetuses with gastroschisis as a consequence of this inflammatory process [14]. Animal research conducted has aimed at reducing the inflammatory effects on the bowel in utero, including amnioexchange or intra uterine coverage of the defect [15]. Additionally, irritation of the bowel due to foreign material such as the silo, might also cause ileus. Ram et al. and Ramadan et al. found that infants with silos have elevated CRP levels during the first week of life, which does not correlate with infection [16, 17]. This could be a possible explanation as to why those who undergo primary closure fed sooner than silo placement in previous studies. Therefore, minimizing the number of days the intestines are exposed to the silo might decrease bowel irritation, subsequently leading to earlier enteral feedings. 

There are currently two common strategies for definitive closure of gastroschisis, primary or silo closure. Eggink et al. found in a single institution retrospective study that using a silo reduction method was associated with longer ventilator times, as well as longer time to first and full feeds [18]. Driver et al. also observed this with surgically placed silos [19]. In contrast, Owen et al. found that there was no difference in the initiation or progression to full feeds between silo and primary closure and that ventilator times were significantly less in the silo placed group [20]. In a meta-analysis to determine closure outcomes, Kunz et al. found that silo closures were associated with better outcomes when stratified to studies with less selection bias [21]. While overall, it appears that those with silo closure do better in the meta-analysis, the slower progression to enteric feeds was consistent throughout many studies [18, 21]. It is possible that the discrepancy between initiation of the feeds has to do with continued irritation to the intestines while in the silo or degree of inflammation present at birth. While we did not reach statistical significance to determine an ideal closure date, the trend was towards closure on or by the fourth day of life. Based on this result, when possible, more aggressive reduction of abdominal contents either with more frequent reduction or possible sutureless closure could be considered to close the defect sooner. At our institution, reductions are done twice daily as tolerated. A more aggressive reduction, possibly three times daily could be considered, with close monitoring for abdominal compartment syndrome with aggressive reduction.

Gestational age at birth has been a commonly debated issue. In our cohort, we found median gestational age to be 35 1/3 weeks, we had one delivery as late as 39 weeks, but all others were delivered by 38 weeks. Within the last two years, conflicting reports have been published: In a 2013 study, Baud et al. showed that outcomes were improved for those induced before 37 weeks, citing less incidence of intestinal atresia, necrosis or perforation [22]. Baud found a trend toward earlier enteral feeding for those induced by 37 weeks, but this was not significant. However, in a 2014 study, Overcash et al. found increased adverse outcomes in those induced before 37 weeks, and found that there was no difference in initiation of oral feeds [10]. Additionally, Cain et al. found there was a higher cost and number of hospitalized days for those delivered before 37 weeks [23]. It is well known however, that careful monitoring in the third trimester should be done due to the known risk of fetal complications in utero [24]. In our study, when comparing those born before and after 37 weeks gestation, we found similar results, with no difference between the groups in relation to initiation of enteral feeds. 

While our study does provide evidence that earlier closure after silo placement correlates with earlier initiation of enteral feeds and achievement of full feeds, there are some limitations to the study. For one, our study is retrospective in nature, and a prospective study could better delineate the timing of closure to feeding. Our data showed a higher rate of preformed silo usage than previously reported, ranging from 50-84.4% [10, 25]. This is possibly due to surgeon preference at initial presentation. However, our findings can be used to guide management of patients who have undergone silo placement. A multicenter collaboration could lead to a larger sample size, to better delineate the ideal closure date after preformed silo placement.

## Conclusion

In conclusion, our study demonstrates that earlier closure of gastroschisis after silo placement was associated with earlier feed initiation and shorter time to full feeds. Returning bowel into the abdominal cavity sooner may minimize intestinal inflammation, leading to earlier return of bowel function. 

## Footnotes

**Source of Support:** None

**Conflict of Interest:** None

